# “Old” and “new” contaminants and their management: learning from the past, looking to the future

**DOI:** 10.1007/s10653-021-01042-6

**Published:** 2021-08-09

**Authors:** Gillian Gibson, Andrew Cundy, Nswana Kafwamfwa, Alex Stewart

**Affiliations:** 1 Gibson Consulting and Training, Tarporley, Cheshire, CW6 0JH UK; 2 School of Ocean and Earth Science, University of Southampton, Southampton, SO14 3ZH UK; 3 Zambia Agriculture Research Institute, Mochipapa Regional Research Station, Choma, Southern province Zambia; 4 College of Life and Environmental Sciences, University of Exeter, Exeter, EX4 4RJ UK

**Keywords:** Emerging contaminants, Human health, Risk, Plastics, Pharmaceutical residues, Antimicrobial resistance

## Abstract

Within the 50 year lifetime of the Society for Environmental Geochemistry and Health (SEGH), we have seen a number of contaminants transfer from being the wonder chemical of their day through to becoming current contaminants of concern. This is also true for a variety of emerging contaminants such as plastic microbeads, pharmaceutical residues, and fire retardant chemicals, amongst others. This thought piece discusses the risk associated with a range of these emerging contaminants, their global nature, how existing models and frameworks can be applied to deal with their impacts, and research and management gaps and challenges.

**Graphic Abstract:**

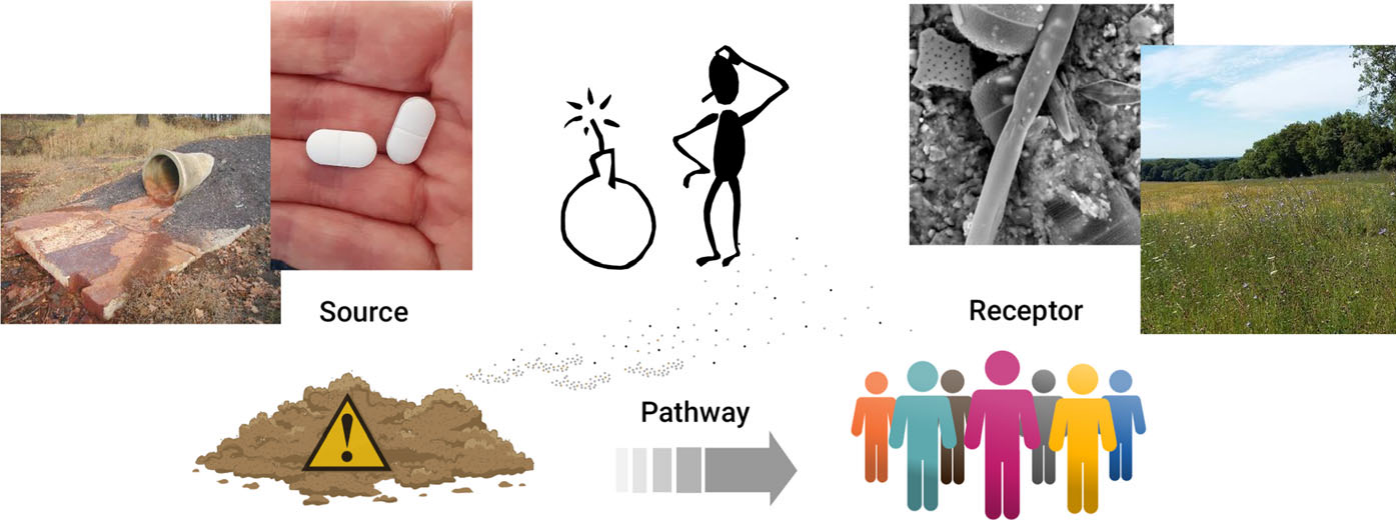

## Introduction

In this 50th anniversary year of the Society for Environmental Geochemistry and Health (SEGH), the environment and human society are faced with a range of global challenges, from global heating to post-pandemic recovery. In parallel, as part of the post-1950 “Great Acceleration” (the dramatic, continuous and roughly simultaneous surge in growth rate across a large range of measures of human activity), we are seeing increased demands on natural resources. This includes so-called critical metals, used in a range of rapidly emerging industrial sectors such as renewable energies. Consequences include a change of geochemical cycling and transfer of these and other metals, and the increased development, use and release of new chemical compounds. Many new compounds are persistent and potentially bioaccumulative in the environment—so-called emerging contaminants.

SEGH itself has a long history of engaging with global issues at the interface of geochemistry, the environment and human health, such as the health risks of lead in soils, and arsenic in drinking waters. In this thought piece, we take a chronological approach, giving an historical perspective on our understanding of risk from both longer-recognised, or “old”, contaminants (such as lead and arsenic) and from emerging, “new”, contaminants (such as plastics and anti-inflammatory drugs), examining the journey to “now” (including development of international management frameworks, and frameworks for risk understanding and assessment), and assessing future trends and challenges. In particular, we focus on the risk associated with a range of emerging contaminants, how existing models and frameworks can be applied for dealing with these risks, and research and management gaps and challenges.

## “Old” and “new” contaminants

One contaminant with which readers of EGAH will be familiar (and to which the Society has made important contributions) has been with us since the early stages of metal exploitation and use: lead. The toxicity of lead has long been recognised, as has that from a range of other metals and metalloids (such as arsenic, cobalt, chromium), with limits imposed on their use and, from both environmental and health viewpoints, their acceptable concentrations in waters, soils and other media. While we understand much about such “old” contaminants, much still remains to be learnt through constantly improving technology which enables measurement of differing chemical forms of an element—speciation—and allows better understanding of the mobility, toxicity, origin or pathway of such an element.

Equally, as we increasingly manage the risk from these elements, and reduce or replace their use, we need to ensure we do not create new risks or problems. Taking mercury out of switchgear, where it did an excellent job as an insulating material, replacing it with a less satisfactory substance, sodium hexafluoride (Ottersbach, 2019), created a completely different problem: widespread release of a gas which is a major contributor to global heating. This example is one of many which highlight the adverse side of the law of unintended consequences. Although referring to economics, the following quote from the French economic journalist Frédéric Bastiat (1801–1850) distinguished between the “seen” and the “unseen” (Bastiat, 1848):

There is only one difference between a bad economist and a good one: the bad economist confines himself to the visible effect; the good economist takes into account both the effect that *can be seen* and those effects that *must be foreseen*. (emphasis added)

It can equally apply to environmental consequences.

However, it is a third effect, the unforeseeable and unanticipated consequences, which are arguably even more important, and which we need to recognise when they occur (de Zwart, 2015). An example would be the fact that recycling is seen to be a good thing. Certainly, from the conservation of raw materials and the avoidance of land contamination this is undoubtedly the case. However, the presence of flame retardants in children’s toys which have been manufactured from recycled plastic, probably from WEEE (waste electrical and electronic equipment) (Turner et al., 2021), is an unanticipated consequence—though it could be argued that insufficient risk assessment had been applied to the process, and the consequences should have been foreseen.

Within the lifetime of SEGH, we have seen some contaminants evolve from being the wonder chemical of their day through to becoming current contaminants of concern. Well-known examples are DDT, chlorofluorocarbons, neonicotinoids and glyphosate, the latter two of which are still controversial. This evolution may be due to technical advances in our ability to detect and track inorganic and organic contaminants in the environment or biological systems at trace (but still potentially toxic) levels, and/or time taken to observe or recognise their wider ecosystem or health risks. There will always be such emerging contaminants, which in time become old or established ones. The battle is how to prevent novel entities such as plastic microbeads, nanoparticles and new chemicals such as fire retardants from becoming persistent problems of the future. As an example, the heat-resistant “forever chemicals” PFOS (perfluoro-octane sulfonate), PFOA (perfluoro-octanoic acid) and related per-fluorinated substances have been widely used in stain-resistant and non-stick coatings, and fire fighting foams. PFOS has been prohibited in the European Union since 2008. Falling serum levels in the USA indicate that exposure to these “forever” chemicals can be reduced (ATSDR, 2020) though they remain chemicals of concern in the UK (Public Health England, 2018a). Despite being chemicals in Annexe B of the Stockholm convention (discussed below), they continue to appear in consumer products, most recently in cosmetics (Whitehead et al., 2021) and remain of global concern. Furthermore, the safety of their replacements is now being challenged (Minet, 2021).

## The journey to “now”

### Global protection and cooperation

The formation of the United Nations post-World War Two enabled the development of internationally binding treaties, for the protection of all. The simultaneous creation of the World Health Organization (WHO) enabled research to be carried out which considers the health of people at a global level, and to inform the development of appropriate treaties. Perhaps the most notable and best known is the Montreal Protocol of 1985, banning the production of chlorofluorocarbons (CFCs) for their ozone-depleting actions, and to which have been added a number of other ozone-depleting substances. For the purpose of this paper possibly the most relevant treaty is the Stockholm Convention (United Nations, 2019). This bans or restricts the production and trading of existing chemicals which are known to have an impact on human health.

Under the European REACH (Registration, Evaluation, Authorisation and Restriction of Chemicals) regulations (European Commission, 2006), European countries aim to implement the UN “Precautionary” (United Nations Global Compact, 2000) and “Polluter Pays” (OECD, 1992) principles: REACH requires the producer of novel (i.e., new and unproven) chemicals to register them before they come to market, and demonstrate that they do not cause harm, either to the wider environment, or to human health. This is a reversal of previous approaches, whereby chemicals were freely traded, and have proved difficult to remove from circulation despite there being ample evidence of their damaging nature.

Each UN convention (or protocol—the term is used interchangeably) is overseen by its own committee. The committee comprises parties, that is, representatives from member states or countries which are signatories to the convention. It is worth noting that three committees of the parties, Rotterdam (importation of hazardous chemicals), Stockholm (restriction of production and use of persistent organic chemicals) and Basel (restriction of movement of hazardous waste between countries), recognise the synergy which exists between the three conventions, and that joint meetings can bring great benefits (Synergies, 2018, and linked webpages). Recommendations from the committees are discussed at the “conference of the parties” which are held on an annual basis: perhaps the most widely recognised recent one (UNEP, 2015) is COP21 which gave rise to the Paris agreement on limiting global temperature increase.

### Monitoring and modelling pollutant migration

The “source, pathway, receptor” model is often used within the environmental community, including relevant industry, to evaluate the risk to the environment (or human health) which may result from loss of control of a substance during the manufacturing process, or following its release to land or water in the contaminated land sector. However, it is a model which is increasingly being deployed to backtrack anthropogenic materials found throughout the environment, acting as a detective to determine the point of production or release, and thus the polluter responsible for creating a problem.

Anthropogenic substances migrate from the points of manufacture, use, and disposal, and are now found all across the globe (Jamieson et al., 2019), from the deepest ocean trenches to the remote poles. They pose a continuing threat to the health of most species including humans, as many continue to bioaccumulate (become concentrated within living organisms) and biomagnify (become more concentrated higher up a food chain). Microplastics, which carry endocrine disrupting chemicals, have been found in the placentas of humans, and have migrated to the foetal side (Ragusa et al., 2021), while engineered nanoparticles (used in hundreds of consumer products, finding increasing use in food industries and with potential for improving pharmaceutical treatments) are capable of crossing cell membranes and inhibiting cell function, or accumulating in organs (including the brain), resulting in neurotoxicity, pulmonary toxicity, vascular dysfunction, genotoxicity, and immunotoxicity (Wang et al., 2021). In the human body, endocrine disrupting chemicals and air pollution particles may act as obesogens, amongst other health effects (de Cock & van de Bor, 2014; Yang et al., 2019)—see Case Study 1: Plastics.

Chemical transformations occur naturally in the environment. This has happened throughout Earth’s history, for example rain falling through the atmosphere, picking up gases as it passes, creating acid rain. However, unlooked-for chemical reactions arise from the interaction of anthropogenic chemicals with the “wild” environment. Well-known examples are the interactions between chlorofluorocarbons (CFCs) and ozone in the stratosphere leading to ozone depletetion, and the diurnal reaction between NOx and polycyclic aromatic hydrocarbons (PAH) creating photochemical smog (Scorer, 1990). It is important to recognise that chemicals which may be developed in the future may initially appear to be benign, but the potential to hybridise into something altogether undesirable cannot be ignored.
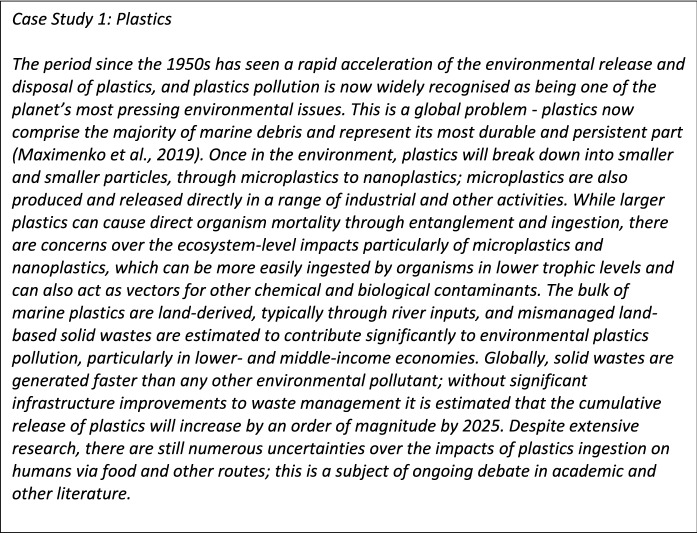


Not all problematic substances have their origins as chemicals designed to impact the wider environment, but may be focused, with the intention of improving human and animal health. Widely used anti-inflammatory drugs such as diclofenac (See Case Study 2: Diclofenac) illustrate the complex interaction of source, pathway, and receptor, acknowledging that pathways may be a receptor in their own right, and that pathways may be multiple, complex and varied.
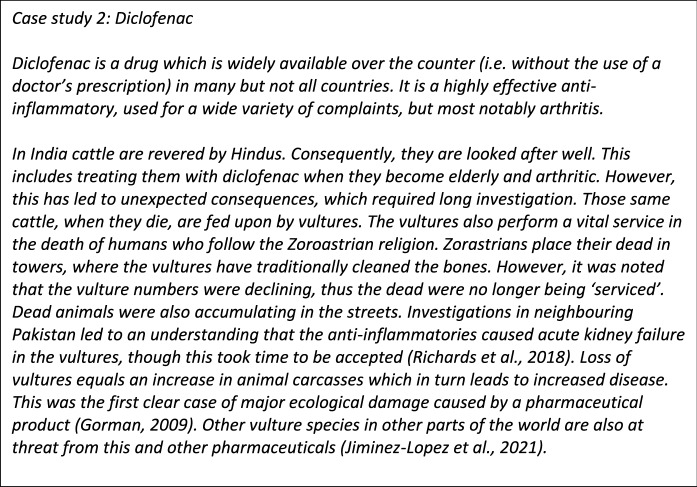


### Wider environmental impact

There is also an impact on plant health resulting from the human and veterinary use of pharmaceutical products (Schmidt & Redshaw, 2015). Anti-inflammatories are excreted by humans and animals, in urine, either directly to groundwater, or via sewage treatment works into wider water bodies. River estuaries around the UK have recently been found to be carrying a heavy load of ibuprofen and related painkillers (University of Hull, 2019). The same applies to the estuary of the Elbe, in northern Germany (Wiegel et al., 2004). These are not alone in being impacted by pharmaceutical materials (Pait et al., 2006). Water used for plant irrigation may be the carrier of these drugs directly to the plants, or the water may be derived from natural uptake via groundwater. The plants are able to accumulate various pharmaceuticals (Herklotz et al, 2010). The failure to provide protection for the environment may have knock-on effects with stunted crops providing less food, and that which is available being contaminated with pharmaceutical residues. Further, the overuse of antibiotics in livestock farming contributes to an increasing number of microbial species resistant to antimicrobial control, generating further risks to animal and human health. See Case Study 3: Antimicrobial resistance (AMR).
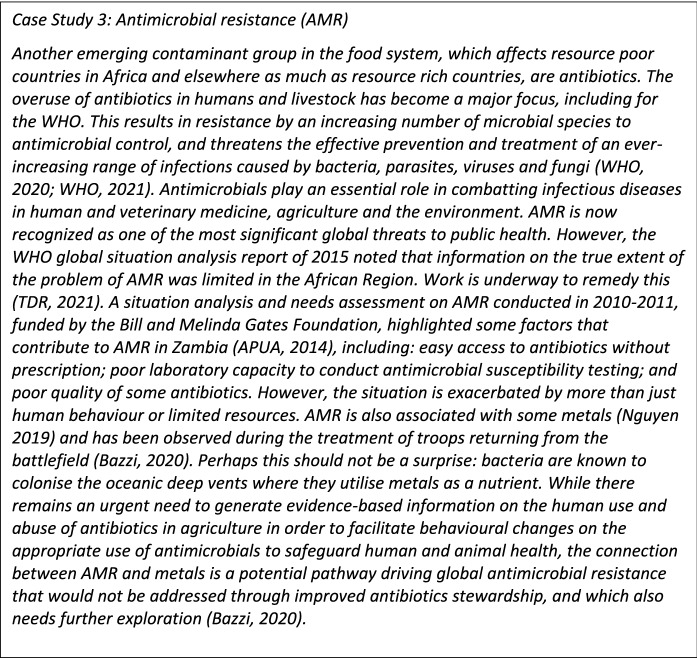


Sewage treatment works were developed for the prevention of faecal material entering waterways. There is nothing within the system which prevents the migration of pharmaceutical residues, nor a range of other chemical residues arising from direct excretion or manufacturing processes. Indeed, the sewage treatment works provide the very conditions for spontaneous chemistry to occur: large vats of diverse material being mixed, in a warm setting, in the presence of bacteria. Further, not all communities have the luxury of the control of waste materials. Many people still live without a functioning latrine; some chemical industries discharge waste directly into nearby streams and rivers. These wastes find their way into groundwater, aquifers, and potentially into the ocean.

While a range of compounds are relatively mobile and persistent, entering marine systems, some of the drugs adhere to sediment. Examination of sediment cores has demonstrated the presence of a wide variety of anthropogenic chemicals adhering to sediment at any one time. Their mobilisation and desorption is dependent upon the chemical under consideration, sediment composition, and the various physicochemical, and biological, processes occurring on and within the sediment. The need for understanding of this interplay between the aquatic environment and geological surfaces is of importance for the health of species which dwell and feed in sediment, and those which predate upon them.

### Consideration and management of risk

**Fig. 1:**
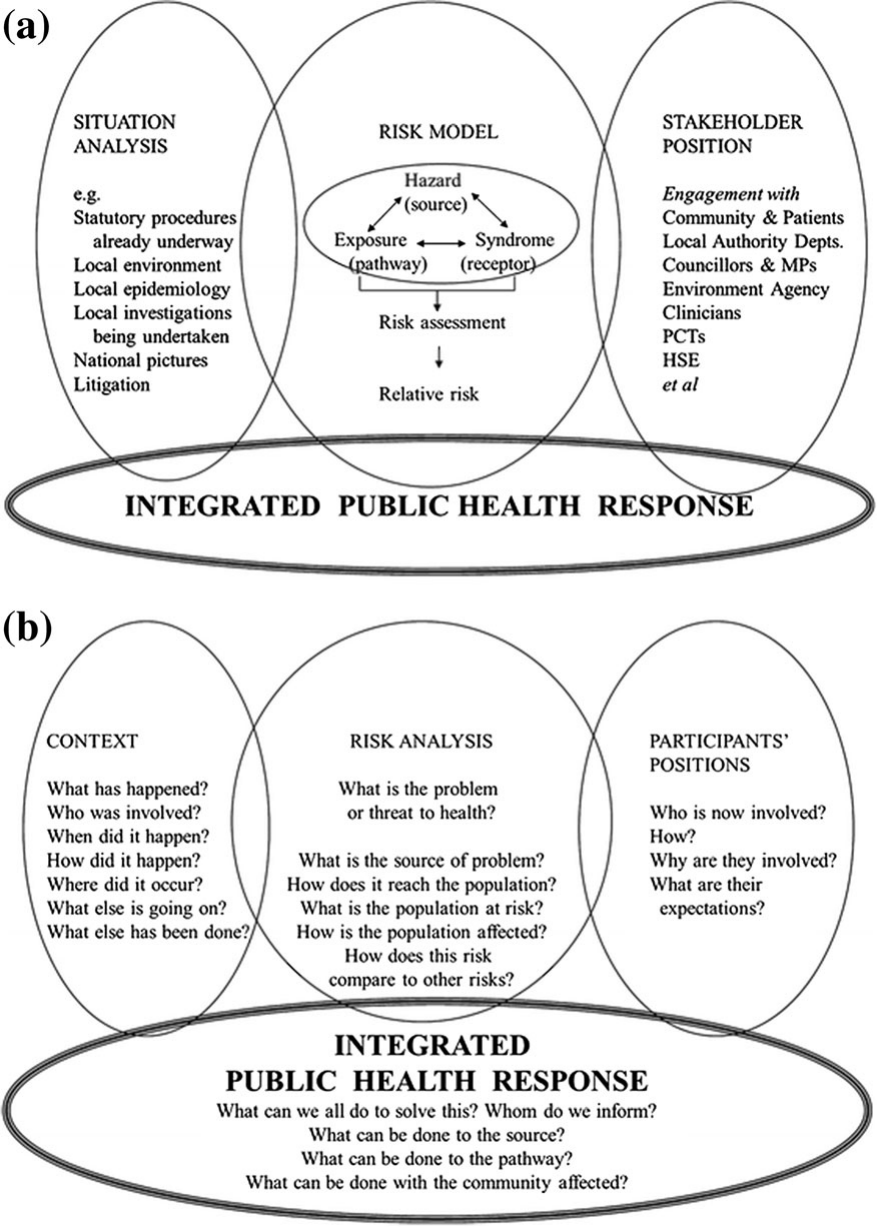
Integrated Public health risk assessment and response. **a** Outline risk assessment. **b** Application: questions to help the practical application. Source: Mahoney et al., 2015; used with permission

It is this uncertainty of material, and unpredictability of outcome, which requires a coordinated approach to protection of human health, such as that used successfully in the North West of England (UK) for two decades or more (Mahoney et al., 2015) (Fig. 1). At its heart, this model again makes use of the source-pathway-receptor model, which is essentially focused on the environment, but overlays it with the human health impact, of hazard, exposure, syndrome.

The WHO has stated that almost a quarter of human disease globally has an environmental cause (WHO, 2006). It can be seen from the model (Fig. 1 (cf. Figure 3)) that there are many contributing factors in the field of understanding and prevention of disease. However, there are unseen tensions which the model can help explore: the desire for employment, job creation, and for business to make money. When a business can see the opportunity to make money, it may be at any cost, including that of its workers. Historically, it has been well-documented that workers exposed to phosphorous in match factories suffered from “phossy jaw”, asbestos exposure has led to mesothelioma across many occupations such as construction workers and dockers, and silicosis and pneumoconiosis affect miners. Improved working conditions responding to legislation have either eradicated these conditions (phossy jaw) or reduced exposure (asbestos, silicosis, pneumoconiosis) in some areas (Stewart, 2020).

Vigilance is required by all involved parties to ensure that emerging industries and technologies do not replace historical problems with fresh ones, that the “polluter pays” principle is embedded within planning, and that there is not a presumption of jobs at any cost and associated impact—neither to the workers themselves nor to the wider public.

**Fig. 2:**
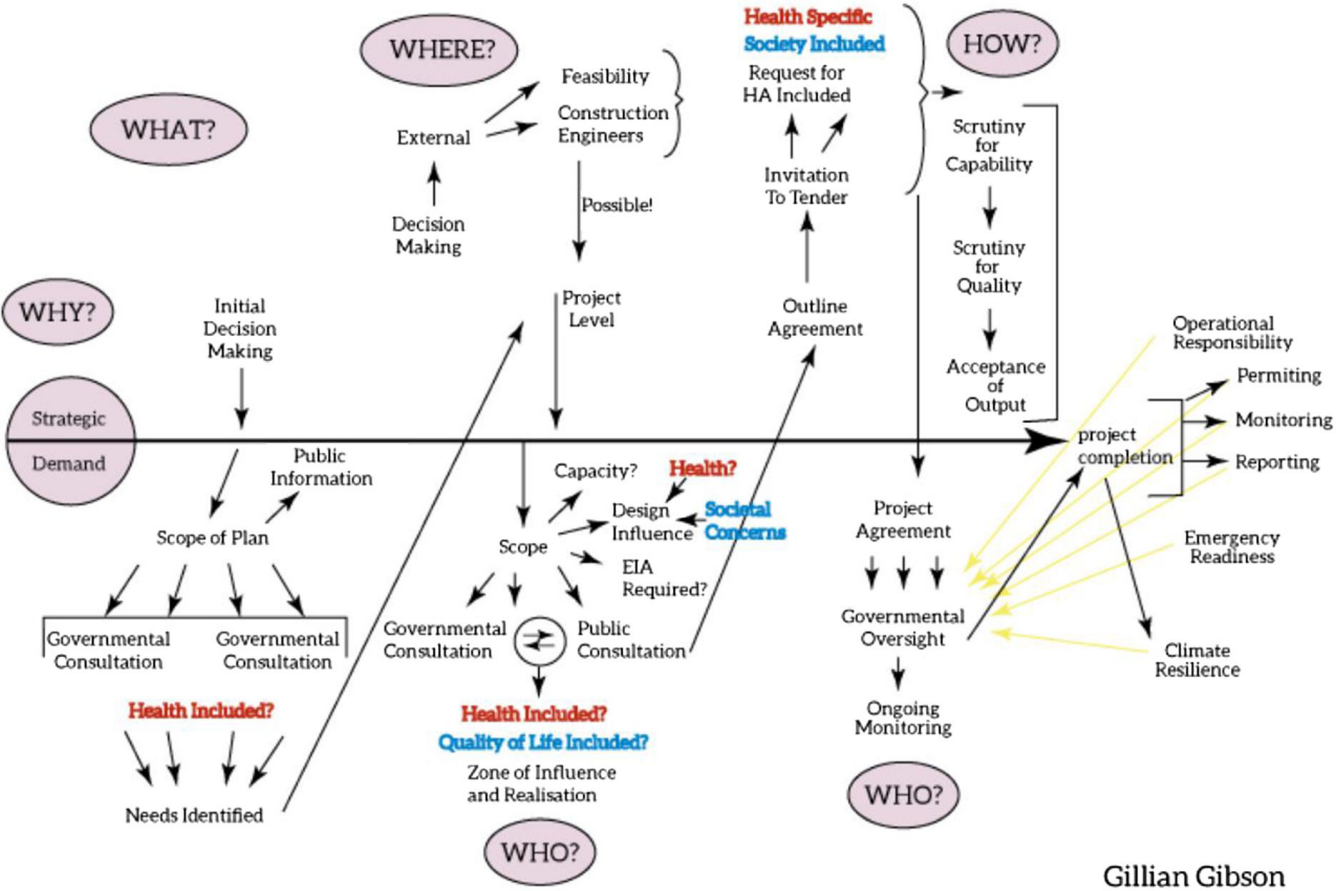
Evaluation of the impact on health from strategic decision-making, through planning, to operational control and decommissioning. Gibson, G. Author, this paper

These principles are embedded in the process of Health Impact Assessment. Understanding that a coordinated approach is required to prevent pollution is at the heart of Gibson’s model (Fig. 2). Evaluating the impact on health along the process of strategic decision-making, through planning, to operational control and finally decommissioning is essential if future contamination is to be prevented.

For example, in order to have a strategy for clean air, a strategic decision to manufacture electric vehicles would recognise that they rely on battery power. This in turn is likely to require several battery manufacturing sites, as well as car manufacturing sites. The batteries require lithium, cobalt and other elements. The mining of these elements will have an impact on the miners, and on the communities where the mining takes place (Entwistle et al., 2019). Some of these will be positive (employment) and some will be negative (ground contamination, inhalation).

Transport of the mined materials will have an environmental impact at a global level, contributing to global heating. Construction and deployment of the manufacturing bases will impact the local environment—again, there will be positive and negative consequences. As the batteries and vehicles are in use, and when they become spent, there will be further impact at a societal level. What happens to the batteries? Are the incorporated metals retrieved and recycled in order to prevent them being released into the wider environment, (with further requirements for a recycling plant, with associated local planning) or are they lost from sight? Currently, a range of rare and/or valuable metals are locked up in unused mobile phones, meaning that further material needs to be mined to fill the gap. Global thinking, cooperation, strategy and policy are required to ensure that the wunderkind of today is not the contaminant of the future (Pagano, 2016).

Since the industrial revolution, the demand for jobs has been an over-riding one. As society observes the damage which has accrued through this approach, with the disadvantage of that approach not always falling on its beneficiaries, there is a strong demand for accountability and cleaner manufacturing. This is manifest as investors seek to divest from traditional “dirty” industry. But it should be remembered that just because something appears to be clean does not mean that it is benign.

There is an additional concern. As high-income countries seek to clean up and operate more effectively, illegal transfer of WEEE to countries with a less well-developed or robust legal system creates an impact in a new region causing new environmental diseases, such as in Agbogbloshie in Ghana (The Guardian, 2014), where previously pristine wetlands are now polluted beyond recognition.

Dismantling of WEEE and burning of cables in order to retrieve valuable metals has led to an environmental catastrophe; fire retardants, products of combustion (dioxins and similar) and metals are released in an uncontrolled manner. The lives of all the residents are impacted due to the contamination of their crops, and the acrid air from the burning of the waste. Those who carry out the “recycling” have increased risk of cancers and other diseases following exposure to a toxic mix of chemicals (WHO, 2015). These recycled materials are then the very ones which find their way into children’s toys, plastic plates, and other consumer goods.

## Future actions and cooperation

Human health itself is a complex response to an uncertain set of parameters: at its most basic, each person is a novel entity by virtue of their genetic make-up. We are still only just touching the surface of our understanding of epigenetics (Carey, 2012). Consequently, understanding our relationship to our wider environment is challenging and evolving.

**Fig. 3:**
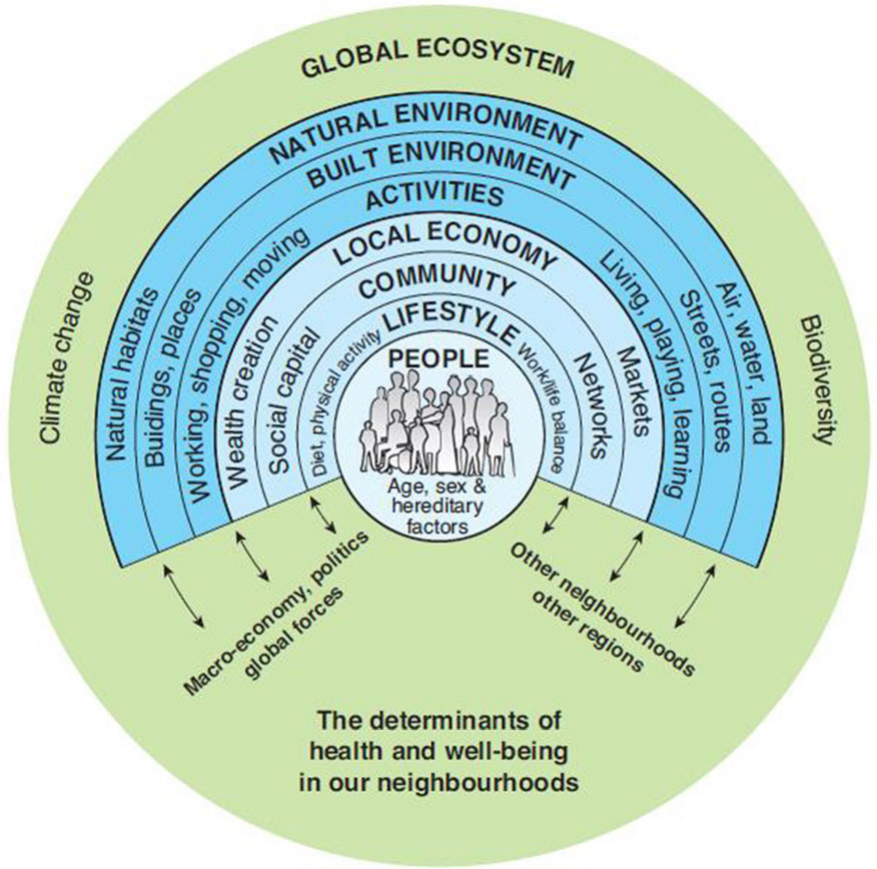
Determinants of health and well-being from the individual level to the global (Barton and Grant, 2006, reproduced with permission)

Barton and Grant (2006) enhanced the original onion skin model (Dahlgren & Whitehead, 1991) which recognises that while people are at the centre of their own lives, their wider environmental and social conditions also influence their health, often with little personal control (Fig. 3). Accordingly, we need to ensure that the environment is as benign as possible, and that it is health-giving rather than a source of ill health.

The only effective way to provide protection for all people and animals is to prevent the escape of future designer chemicals, wastes, residues, etc., into the environment. Before chemicals, of whatever sort, are released into the environment, we need to be sure that a future “wicked problem” is not being created. A wicked problem may be considered to be a complex problem with no clear right or wrong, good or bad answer and which changes as we engage with it. It needs recognition in the first place as such a class of problem, and then it needs a multi-agency or multi-professional approach, with a willingness to observe continually the problem and adapt the response as appropriate until a satisfactory result is reached or the problem is recognised as beyond our ability to answer. If the latter, the problem may require disaggregation into smaller problems, which can be addressed more effectively (Mahoney et al., 2015).

For this to happen, wider cooperation between the various disciplines, such as already occurs between researchers associated with SEGH, needs to become the norm, in order to foster greater understanding of the risks and opportunities. The risks need to be communicated clearly to policy makers and funders to inform sound decision-making.

Longitudinal health studies are difficult to conduct, but are an excellent way to be sure of identifying adverse effects which may take many years to emerge. Sometimes they happen accidentally (“natural experiments”, such as the follow-up of the cohort exposed to radiation as a result of the disaster at Chernobyl, Hatch & Cardis, 2017). Longitudinal studies are important not only to observe effects on the people exposed to a variety of environmental contaminants, but also on the following generation. A female foetus develops all the eggs while in utero, it subsequently becomes fertilised when the female is of reproductive age. Exposure in utero to environmental pollutants may have an effect some forty or more years later as the next generation (possibly two) reaches maturity, exhibiting complex and poorly understood syndromes, when contemporary physicians may not necessarily be aware of historical issues. There is also (as yet) limited evidence of transfer of epigenetic impacts through four generations of male progeny (Weinhold, 2006). Clearly prevention is better than cure.

In the wider environment, top predators bioaccumulate contaminants with catastrophic results. Lactating females pass these contaminants to their offspring in their milk. Orca calves are thought to have died as the burden of PCBs in the maternal milk is too contaminated for them to process (Desforges et al., 2018). This is a very visible problem as cetaceans wash up on beaches and can be subject to post-mortem. It may be happening elsewhere in the natural world but less visibly. Domesticated animals are likely to be protected from some of the problems as they are fed regulated food. Wild animals may be exposed to a cocktail of pollutants which have a subclinical effect but which, in the fullness of time, can show substantial impact, as they transfer across the generations. Longitudinal studies on animal cohorts may help to unravel the aetiology of environmental impacts which perhaps give rise to human health problems.

## Discussion: for the future—synergies and trade-offs

In science, there are many synergies and trade-offs. As an example, the fight against global heating is driving a global move away from fossil fuel use. This is significant as currently, at both national and international levels, the human population and the rest of the global biota are exposed to poor air quality derived from burning of fossil fuels, whether for heating or transport (WHO, 2013). Particulate matter less than ten microns in diameter (PM_10_) is known to enter the body and cause damage to the circulatory system. Smaller particles of 2.5 microns (PM_2.5_) are able to enter the blood stream and travel around the body: they are associated with miscarriages, early birth, low birthweight, Parkinson's disease, stroke, infertility and diabetes type 2. The smaller particles of only one micron (PM_1_) are known to cross the blood brain barrier and have been implicated in Alzheimers and other neurological diseases (Ascher et al., 2017; Finch, 2018). A permanent shift away from fossil fuel will drive a reduction in fossil fuel-derived fine particles, though there will still remain anthropogenic particulates from the breakdown of vehicle parts (brakes, tyres, windscreen wipers), dust from mining and construction activities, and other (including natural) contributions to particles and aerosols. All of these will need to be managed at a global level if human and other biotic health is to be protected.

Similarly, the changed pattern of behaviour at national and global levels due to the Covid-19 pandemic has provided the opportunity to observe changes in air quality resulting from altered (reduced, by and large) patterns of manufacturing and travel. The requirement for wearing of masks to prevent inhalation of viral aerosols may also be providing protection from more general air pollution. However, spending a greater amount of time in our homes, where masks are not being worn, is likely to be exposing us to a different set of contaminants indoors. Indeed, indoor air may pose as many problems as outdoor air. Harrad and colleagues have been studying household dust for several years and found it to be contaminated with fire retardants (Tao et al., 2016). These are emitted from electrical equipment, furnishing, and carpets, to which the fire retardants (linked to endocrine disruption, developmental issues, cancers and other adverse health effects) have been applied. A paradox is embedded here; it may not be possible to see the results of either of these changes as they are masked by the adverse health effects of the pandemic virus itself.

Not all air pollution is anthropogenic in nature; volcanic eruptions for example also contribute to poor air quality, as do natural dust storms. Humans have no direct control over these events, though the health impact can be large. A combination of anthropogenic and naturally occurring particulate matter has the potential to cause long lasting health impacts (Citris Policy Lab., 2019), and also impact food and other crops—the load of particulates contributes to a reduction in photosynthesis, with an attendant reduction in biomass. This may become critical in crop production (Prajapati, 2012). Additionally, aerosols, whether naturally occurring (e.g. generated by wind passing over the ocean, particles picked up by mists) or human-induced (e.g. crop spraying, vapour from unburnt aircraft fuel), contribute to ill health by enabling intake of mixed material into the body. A mixture of volatile organic compounds, metals, and carbonaceous material all take their toll on the body, at a cellular level as well as a system level (Pardo et al., 2020). Increasingly, the awareness of the metalliferous content, and chemical reactivity of the aerosols both within the aerosols themselves and then within biological systems, indicates a need for a coordinated approach to control of emissions, in addition to a global responsibility for end-of-life action on waste (Despeisse et al., 2015).

## Concluding thoughts

Consideration of the multiple determinants of health (Fig. 3) in conjunction with some of the issues raised here demonstrates the need for evaluation of the effect of environmental exposure, whether to deliberate or accidental pollutants, on the health of humans: (a) from preconception to senescence; (b) in the home, workplace, and wider world; (c) in the impact on the food and water they consume; and (d) in and through the very air they breathe. The use of the source-pathway-receptor approach needs to be considered across time and space as well as in close proximity and immediacy.

Furthermore, two complementary concepts need to be kept in mind, (1) One Health (WHO, 2017) and (2) an all hazards approach. One Health is an approach to designing and implementing programmes, policies, legislation and research in which multiple sectors communicate and work together to achieve better public health outcomes (cf. the approach in Fig. 2 and related text). It includes the responses to address the challenge of AMR, made more urgent by the recognition of environmental inputs to AMR (Case Study 3). As such, One Health goes beyond the WHO (2017) description of infectious diseases since environmental pollution affects more than just humans. The all hazards approach (Norris et al., 2020) is still developing, but aims to replace the long-standing reductionist focus on individual hazards with a broad view across disciplines, factors and issues to the commonalities in understanding and response.

In this article, we have described the effects on the global environment which may be attributable to pollution. There is a financial cost associated with these negative effects. The UK government through Public Health England have estimated the costs to the health service of poor air quality (Public Health England, 2018b). For 2017, it was estimated to be £42.9 million, and considered to be reaching £5.3 billion by 2035, unless action is taken. That is one country, one pollutant group. At a global level Cohen (2017), arguing cogently for investment in sustainability management to reduce the costs of pollution, quoted The Lancet Commission on pollution and health (Landrigan et al., 2017):

*In 2015, diseases caused by air, water and soil pollution were responsible for 9 million premature deaths, that is 16% of all global death. Exposures to contaminated air, water and soil kill more people than smoking, hunger, natural disasters, war, AIDS, or malaria.*

This eclipses the number of deaths caused by the Covid pandemic to date. The Environmental Protection Agency (EPA) of America (EPA, 2011) has indicated that return on investment to prevent pollution is at a rate of thirty to one. Similarly, WHO (2010) notes that the cost–benefit from reducing lead hazards in the USA outweighs that of investment in vaccines, which have long been described as the single most cost-beneficial health intervention, by a factor between 17 and 220. The question needs to be posed that with such rates of return, why isn’t everyone investing? If that return was to appear in the portfolio of an investment company investors would be clamouring for a share.

There are protocols in place which have global agreement through the United Nations and supported by WHO which are intended to prevent trade of harmful substances. The Sustainable Development Goals (SDGs) (United Nations, 2015) are intended to enable nations to trade in ways which do not negatively impact the environment and require good governance at a business, government and personal level. For both “old” and “new” contaminants, the global sustainability agenda and post-Covid recovery offers opportunities and challenges for managing the health risks of air, soil, sediment and water pollution, and reducing global inequalities in exposure.

Geochemists and health professionals in SEGH, and beyond, need to keep warning of consequences of unregulated chemicals. Biologists and health professionals need to keep warning of the implications of the use of untested materials. More data, including robust, comparable, measurements of environmental and other media (at often very low concentrations) are needed to drive more effective policy, regulation and risk management, while also recognising that data interpretation may change with time, and a strong narrative today may be challenged in the light of new information in the future. Key needs here are to provide accessible technology, methods and data to monitor effectively across low and middle income, as well as higher income, countries, and to link data across disciplines. All of us need to collaborate across and beyond our disciplines to inform policy, regulation and practice, and support evidence-based, holistic and integrated risk and response approaches which will benefit the whole planet as well as ourselves.

## Data Availability

All data and material are presented within the publication, or are cited from the referenced literature.
